# Inflammation-associated clinical prognostic factors and primary patency after drug-coated balloon angioplasty for recurrent autogenous arteriovenous fistula stenosis: a single-center retrospective cohort study

**DOI:** 10.3389/fcell.2026.1977789

**Published:** 2026-10-27

**Authors:** Chaojiang Su, Chengwen Dang, Huan Wu, Yan Jiang, Yiping Yan, Lizhu Wang, Guanglei Chen, Liting Liu, Yan Chen, Zongyang Liu, Bin Zhao, Yunzhi Chen

**Affiliations:** 1 Guizhou University of Traditional Chinese Medicine, Guiyang, Guizhou, China; 2 Department of Nephrology, The Affiliated Cancer Hospital of Guizhou Medical University, Guiyang, Guizhou, China; 3 Department of Nephrology, Xining Second People’s Hospital, Xining, Qinghai, China; 4 Department of Nephrology, Beijing Haidian Hospital (Haidian Section of Peking University Third Hospital), Beijing, China; 5 Qihuang College, Guizhou University of Traditional Chinese Medicine, Guiyang, Guizhou, China

**Keywords:** arteriovenous fistula, drug-coated balloon, inflammation, inflammatory markers, primary patency, prognostic factors, restenosis, retrospective study

## Abstract

**Background:**

Recurrent autogenous arteriovenous fistula (AVF) stenosis occurs in a vascular environment in which injury, neointimal hyperplasia and inflammation may coexist. Whether routine inflammatory or hematological markers contribute to clinical prognosis after drug-coated balloon (DCB) angioplasty remains uncertain. This retrospective study examines clinical outcomes and inflammation-related prognostic associations without assuming that inflammatory pathways were measured or causally established.

**Methods:**

This single-center retrospective cohort comprised 357 patients treated between February 2023 and May 2026. The primary outcome was time from index DCB angioplasty to clinically driven target-lesion reintervention. Kaplan–Meier and Cox analyses used available follow-up; a separate clinical logistic model addressed failure within 12 months in 356 patients with complete model data. No non-DCB comparator or patient-level molecular measurements were available.

**Results:**

Mean brachial-artery flow increased from 288.98 ± 139.37 to 980.92 ± 174.66 mL/min and minimal luminal diameter from 1.52 ± 0.50 to 5.21 ± 0.63 mm (both P < 0.001). Kaplan–Meier primary patency was 95.24%, 84.03%, 68.90%, and 55.40% at 3, 6, 9, and 12 months. In the extended Cox model (338 patients; 191 events), previous PTA count (HR 1.129 per procedure, P = 0.003) and lesion length (HR 1.105 per cm, P = 0.001) were associated with greater hazard; postprocedural flow (HR 0.889 per 100 mL/min, P = 0.014) and red-blood-cell count (HR 0.804 per 10^12^/L, P = 0.027) were associated with lower hazard. The five-fold cross-validated AUC of the clinical 12-month model was 0.706.

**Conclusion:**

In this selected single-center cohort, recurrent AVF stenosis after DCB angioplasty was associated with previous PTA burden, lesion length, postprocedural flow, and red-blood-cell count. Routine blood-cell measures provide an exploratory inflammation-related clinical context but do not establish inflammatory mechanisms or validate a specific inflammatory biomarker. Independent validation and patient-level inflammatory measurements are required.

## 1 Introduction

An autogenous arteriovenous fistula (AVF) provides durable vascular access for maintenance haemodialysis, yet its continued function is threatened by stenosis, impaired access flow, and repeated interventions (Lok et al., 2020; Beathard et al., 2018; MacRae et al., 2016). Clinically significant stenosis is not simply a reduction in vessel diameter: when accompanied by impaired dialysis delivery, abnormal access findings, or haemodynamic dysfunction, it may necessitate another procedure and interrupt treatment (Lok et al., 2020; Beathard et al., 2018). Recurrent stenosis is particularly consequential because patients have already undergone percutaneous transluminal angioplasty (PTA), and successive procedures may be followed by further episodes of access dysfunction. Identifying which patients experience patency loss after treatment is therefore important for follow-up planning. The present study addresses a defined subgroup—patients with recurrent stenosis of an autogenous AVF treated with a drug-coated balloon (DCB)—rather than the full spectrum of dialysis-access disease.

**TABLE 1:** Baseline continuous variables by primary-patency outcome.

Variable (mean ± SD)	Patent (n = 152)	Non-patent (n = 205)	t	P Values
Age (years)	50.32 ± 13.05	50.22 ± 12.50	0.08	0.937
Dialysis vintage (months)	63.55 ± 43.95	62.77 ± 41.64	0.17	0.866
Prior PTA procedures (n)	2.09 ± 1.52	3.10 ± 2.03	5.38	<0.001
Pre-procedural minimal diameter (mm)	1.52 ± 0.50	1.51 ± 0.50	0.18	0.858
Lesion length (cm)	2.90 ± 1.27	3.97 ± 2.32	5.56	<0.001
Pre-procedural brachial-artery flow (mL/min)	291.80 ± 144.16	286.89 ± 136.03	0.33	0.745
Post-procedural diameter (mm)	5.22 ± 0.61	5.20 ± 0.64	0.39	0.694
Post-procedural brachial-artery flow (mL/min)	1000.90 ± 176.98	966.10 ± 171.86	1.86	0.064
Flow gain (mL/min)	709.11 ± 173.10	679.21 ± 177.03	1.60	0.111
Diameter gain (mm)	3.70 ± 0.55	3.68 ± 0.71	0.25	0.802
Red-blood-cell count (10^12^/L)	4.05 ± 0.83	3.87 ± 0.71	2.06	0.040
White-blood-cell count (10^9^/L)	6.55 ± 1.80	6.20 ± 2.04	1.65	0.101
Platelet count (10^9^/L)	192.78 ± 63.74	174.53 ± 69.28	2.52	0.012
Haemoglobin (g/L)	119.66 ± 21.65	116.10 ± 20.57	1.53	0.126
Albumin (g/L)	40.89 ± 4.38	41.12 ± 3.39	0.53	0.599
Creatinine (µmol/L)	1057.51 ± 351.45	1070.28 ± 336.54	0.34	0.732
D-dimer	0.58 ± 0.42	0.82 ± 1.47	1.86	0.065
Calcium (mmol/L)	2.29 ± 0.31	2.24 ± 0.24	1.47	0.143

Data are mean ± standard deviation. Comparisons by independent-samples t-test. PTA, percutaneous transluminal angioplasty. Bold rows denote P < 0.05.

The development of AVF stenosis involves a complex response of the vessel wall to altered haemodynamics and injury. Venous neointimal hyperplasia, extracellular-matrix accumulation, and maladaptive vascular remodelling contribute to progressive luminal narrowing (Brahmbhatt et al., 2016; Lee and Roy-Chaudhury, 2009; Roy-Chaudhury et al., 2006; Roy-Chaudhury et al., 2007; Allon et al., 2013; Kokubo et al., 2009). Inflammation is an important part of this established pathophysiological framework: endothelial perturbation and tissue injury may be accompanied by recruitment and activation of inflammatory cells, cytokine signalling, and interactions between inflammatory and vascular-wall cells (Brahmbhatt et al., 2016; Lee and Roy-Chaudhury, 2009; Roy-Chaudhury et al., 2006). The uraemic environment and repeated cannulation or endovascular manipulation provide additional clinical contexts in which vascular injury and inflammatory responses may coexist. These processes offer biological reasons to study restenosis in relation to inflammation, but they cannot be assumed to have occurred in any particular patient solely because that patient experienced repeat stenosis. Our clinical records did not contain vascular tissue assays, circulating cytokine measurements, or pathway-specific functional tests; accordingly, inflammation is a rationale for the study and an interpretive context, not a directly measured mechanism.

The clinical course of recurrent stenosis may reflect several overlapping influences. Lesion length describes anatomical disease extent; previous PTA count reflects intervention history; and access flow after treatment describes the immediate haemodynamic result. Each can be measured in routine practice and may carry prognostic information without being a direct marker of tissue inflammation. Peripheral blood-cell measures are also readily available and may provide exploratory information about the patient’s systemic condition. For example, leukocyte and differential counts, platelet count, and red-blood-cell indices may vary in patients with chronic kidney disease because of inflammation, infection, anaemia, iron handling, medication, and other factors. Therefore, associations between these indices and patency outcomes require cautious interpretation: a blood-count association does not prove inflammatory causation, and an individual laboratory value should not be labelled an inflammation-specific biomarker without appropriate validation. Prior investigations of access outcomes and routinely collected haematological indicators provide a clinical rationale for exploring these relationships (Zheng et al., 2021; Zhu et al., 2022; Huang et al., 2023; Li et al., 2024), while the wider literature on vascular-access injury supports considering inflammation in the discussion (MacRae et al., 2016; Brahmbhatt et al., 2016; Lee and Roy-Chaudhury, 2009; Roy-Chaudhury et al., 2006; Roy-Chaudhury et al., 2007; Allon et al., 2013; Kokubo et al., 2009).

Balloon angioplasty can restore luminal calibre, but recurrent stenosis may limit the duration of clinical benefit. Paclitaxel-coated DCBs were introduced to provide local antiproliferative drug delivery following lesion preparation, and randomized studies have evaluated their performance against conventional angioplasty in selected populations with dysfunctional AVFs (Lookstein et al., 2020; Holden et al., 2022; Lookstein et al., 2023; Zhao et al., 2024; Trerotola et al., 2020; Pang et al., 2021). Those trials inform the treatment context; they do not establish the comparative benefit of DCB therapy in the present single-arm retrospective cohort. Moreover, outcomes reported in broader dysfunctional-access populations may not transfer directly to patients selected for recurrent stenosis after one or more prior PTA procedures. Within this specific population, it is useful to quantify target-lesion primary patency and examine whether lesion burden, intervention history, haemodynamic response, and routine blood indices are associated with subsequent clinically driven reintervention. Such an analysis can identify prognostic associations, but without a non-DCB comparator it cannot attribute the observed patency trajectory to the drug coating itself.

We therefore conducted a single-centre retrospective study of 357 patients with recurrent autogenous AVF stenosis undergoing DCB angioplasty. Our objectives were to describe immediate anatomical and haemodynamic changes and longitudinal target-lesion primary patency; evaluate clinical, procedural, and haematological factors associated with patency loss; and assess a clinical-only model for failure within 12 months. We distinguish observed prognostic associations from hypotheses about inflammatory biology and do not claim that this cohort establishes a specific inflammatory mechanism or an inflammation-directed surveillance strategy.

## 2 Materials and methods

### 2.1 Study design and setting

This was a single-centre, retrospective observational cohort study conducted at the Department of Nephrology, The Affiliated Cancer Hospital of Guizhou Medical University. Patients who underwent drug-coated balloon angioplasty for recurrent autogenous arteriovenous-fistula stenosis between February 2023 and May 2026 were screened for eligibility. The minimum planned follow-up after the index procedure was 12 months, and available longer-term follow-up was retained for survival analyses, with a maximum observed duration of 42 months. The study was performed in accordance with the Declaration of Helsinki and approved by the Ethics Committee of the Affiliated Cancer Hospital of Guizhou Medical University (Approval No. 2023-06-20). Given the retrospective design and the use of de-identified clinical data, the requirement for individual informed consent was waived.

### 2.2 Participants and eligibility criteria

Adult patients receiving maintenance hemodialysis through an autogenous AVF were eligible when they underwent DCB angioplasty for recurrent, clinically significant target-lesion stenosis after at least one prior PTA procedure. Target-lesion stenosis and lesion location were determined by duplex ultrasonography. A clinically significant restenosis was defined as greater than 50% luminal diameter reduction relative to the adjacent normal vessel together with at least one clinical or haemodynamic indicator of access dysfunction, including reduced access flow, increased venous dialysis pressure, an abnormal thrill or bruit, arm swelling, prolonged bleeding after needle withdrawal, difficult cannulation, recirculation, or inadequate delivered dialysis (Lok et al., 2020).

Eligibility was operationalised as follows. The inclusion criteria were: (1) age 18 years or older and on established maintenance hemodialysis; (2) a functioning autogenous AVF used as the dialysis access; (3) at least one previous PTA procedure on the same access circuit before the index DCB procedure, thereby defining a recurrent-stenosis population; (4) duplex-ultrasound-confirmed clinically significant target-lesion restenosis requiring repeat intervention; (5) treatment of the target lesion with a paclitaxel DCB at the index procedure; and (6) availability of baseline clinical, anatomical, laboratory, procedural and follow-up data sufficient to ascertain primary patency. The exclusion criteria were: (1) arteriovenous graft or other non-autogenous access; (2) isolated central-venous interventions; (3) acute access thrombosis treated primarily by thrombectomy without an underlying treatable stenosis; (4) *de novo* stenosis without previous PTA; and (5) incomplete procedural or follow-up documentation precluding outcome ascertainment. After these criteria were applied to 405 source records, 48 arteriovenous-graft or non-AVF records were excluded, leaving 357 patients in the primary analytic cohort. Of the 357 patients, 356 had complete data for the prespecified 12-month prediction model.

### 2.3 Procedure and periprocedural care

All procedures were performed by experienced nephrologists using a standardised ultrasound-guided endovascular technique. The target lesion was localised and characterised by duplex ultrasonography, and all efficacy measurements were based on ultrasound-derived access-flow and luminal-diameter parameters. After guidewire crossing, the target lesion was initially predilated with a conventional high-pressure balloon. When adequate lesion expansion could not be achieved because of a persistent balloon waist, marked elastic recoil, or an insufficient luminal gain, further lesion preparation was performed using an ultra-high-pressure non-compliant balloon or a specialty lesion-preparation balloon, as appropriate.

Specialty lesion-preparation balloons included cutting balloons, blunt scoring balloons, and sharp scoring balloons. The choice of specialty balloon was made at the operator’s discretion according to lesion morphology, resistance to initial balloon dilatation, and the degree of residual stenosis. These specialty balloons were used as adjunctive devices to modify resistant or fibrotic lesions, improve luminal expansion, and facilitate uniform contact between the subsequently applied drug-coated balloon and the vessel wall. They were therefore considered lesion-preparation devices rather than the index drug-delivery treatment.

After adequate lesion preparation had been achieved, the target lesion was treated with a paclitaxel-coated arteriovenous-fistula high-pressure balloon dilatation catheter (ACOART AVENS®; Acotec Scientific Co., Ltd., Beijing, China; NMPA registration no. 20233030503). The drug-coated balloon was selected to match the reference-vessel diameter, positioned to cover the entire prepared target lesion, and inflated in accordance with the manufacturer’s instructions for use. Paclitaxel delivery during sustained balloon inflation was intended to inhibit vascular smooth-muscle-cell proliferation and migration, thereby attenuating neointimal hyperplasia and prolonging the interval before clinically driven target-lesion reintervention.

For analysis, lesion-preparation balloons were categorised as conventional high-pressure balloons, ultra-high-pressure non-compliant balloons, or specialty balloons. Cutting balloons, blunt scoring balloons, and sharp scoring balloons were pooled within the specialty-balloon category because they represented related adjunctive lesion-preparation strategies rather than separate index drug-coated treatments. This prespecified grouping was used for the descriptive and outcome analyses.

Technical success was defined on the basis of ultrasound and clinical access findings as residual stenosis of less than 30% after drug-coated balloon angioplasty, together with restoration of a palpable thrill and an audible bruit. Clinical success was defined as successful use of the access for prescribed haemodialysis after the procedure without immediate target-lesion reintervention, accompanied by improvement in access-flow or access-dysfunction parameters. Antiplatelet and anticoagulant management, blood-pressure control, and dialysis prescriptions followed routine institutional practice and each patient’s pre-existing treatment regimen.

### 2.4 Data collection and variables

For each patient, demographic, clinical, anatomical, procedural, haemodynamic, and laboratory variables were extracted from the electronic medical record and procedural database using a predefined case-report form. Demographic and clinical variables included age, sex, dialysis vintage, hypertension, diabetes mellitus, and primary renal disease. Diabetes was coded as a binary comorbidity variable and was treated as an adjustment covariate rather than as a presumed protective factor. Primary renal disease was grouped into hypertension-related, diabetes-related, chronic glomerulonephritis or immune-related, gout-related, and unspecified or other categories.

Anatomical and procedural variables included target-lesion location, lesion length, number of previous percutaneous transluminal angioplasty procedures performed on the access circuit, drug-coated balloon device information, and the lesion-preparation balloon category. Lesion-preparation balloons were classified as conventional high-pressure, ultra-high-pressure non-compliant, or specialty balloons. The specialty-balloon category comprised cutting balloons, blunt scoring balloons, and sharp scoring balloons. Because these three specialty devices were used for adjunctive lesion preparation rather than as independent drug-delivery treatments, they were pooled into a single specialty-balloon category for statistical analysis.

Haemodynamic and anatomical efficacy variables comprised brachial-artery access-flow volume and the minimal luminal diameter at the narrowest point of the stenosis, each measured by duplex ultrasonography immediately before and after the index procedure. Flow gain and diameter gain were calculated as the post-procedural value minus the corresponding pre-procedural value. Laboratory variables obtained from the pre-procedural blood sample comprised complete blood count and related indices, including red-blood-cell count, white-blood-cell count, platelet count, haemoglobin, haematocrit, and absolute lymphocyte, neutrophil, and monocyte counts; serum biochemical indices, including total protein, albumin, alkaline phosphatase, urea, creatinine, uric acid, calcium, phosphorus, and potassium; and additional variables including D-dimer, parathyroid hormone, and left-ventricular ejection fraction.

### 2.5 Data cleaning and quality control

Before analysis, the dataset underwent prespecified quality control. Continuous variables were examined for missingness, range errors and physiological plausibility; values outside predefined biological limits were set to missing only for the affected variable and the reason for exclusion was logged. Variables affected by this rule included red-blood-cell count, platelet count, albumin, alkaline phosphatase, urea, creatinine, uric acid, calcium, phosphorus and potassium. One impossible calendar date was corrected to the nearest valid date after comparison with the source record. Outcome dates were not imputed. Complete-case analysis was used for each model, and the effective sample size and number of events were reported for multivariable Cox modelling and for the 12-month prediction model.

### 2.6 Outcomes

The primary outcome was post-procedural target-lesion primary patency, defined as the time from the index drug-coated balloon procedure to the first clinically driven reintervention of the treated target lesion, consistent with dialysis-access patency definitions (Lok et al., 2020). Reintervention was counted when repeat endovascular or surgical treatment was performed because of recurrent access dysfunction attributable to the target lesion. The full-cohort time-to-event analyses used all available follow-up, with a maximum observed follow-up of 42 months. Patients without target-lesion reintervention were censored at the date of the last documented clinical, dialysis-unit, or ultrasound access assessment, or at the database-lock date, whichever occurred first. Death, transplantation, or transfer of dialysis care without target-lesion reintervention was treated as censoring at the last date on which access status could be verified. For the fixed-time prediction analysis, 12-month primary-patency failure was defined as clinically driven target-lesion reintervention occurring within 365 days of the index procedure.

### 2.7 Statistical analysis

Continuous and categorical baseline summaries, paired pre/post comparisons, Kaplan-Meier patency estimates and Cox proportional-hazards analyses were performed. The extended Cox model used 338 complete cases with 191 events. A clinical logistic regression model for the fixed 12-month endpoint included lesion length, postprocedural flow, prior PTA count, platelet count and hypertension; 356 patients with 159 documented 12-month failures were included. Apparent and five-fold cross-validated AUC, Brier scores and calibration measures were reported. No molecular predictor was entered in any clinical model. The source data are required to verify the precise handling of participants censored before 365 days, proportional-hazards assumptions, missing-data sensitivity analyses, nonlinearity and optimism-corrected validation; none of these additional analyses is claimed as completed here.

## 3 Results

### 3.1 Cohort composition and baseline characteristics

Between February 2023 and May 2026, 405 source records were screened. After 48 arteriovenous-graft or non‐autogenous‐AVF records were excluded, 357 patients with recurrent stenosis of an autogenous arteriovenous fistula treated with drug‐coated balloon angioplasty were included in the primary analytic cohort (Figure 1). Of the 357 patients, 356 had complete data for the prespecified 12-month prediction model, of whom 159 (44.7%) experienced primary‐patency failure within 12 months. During the full available follow‐up, 205 of the 357 fistulae (57.4%) underwent clinically driven target-lesion reintervention, while 152 (42.6%) were censored without target‐lesion reintervention. Baseline characteristics by primary‐patency outcome are summarized in Tables 1, 2. The distributions of selected clinical and categorical variables are shown in Figure 2.

**FIGURE 1:**
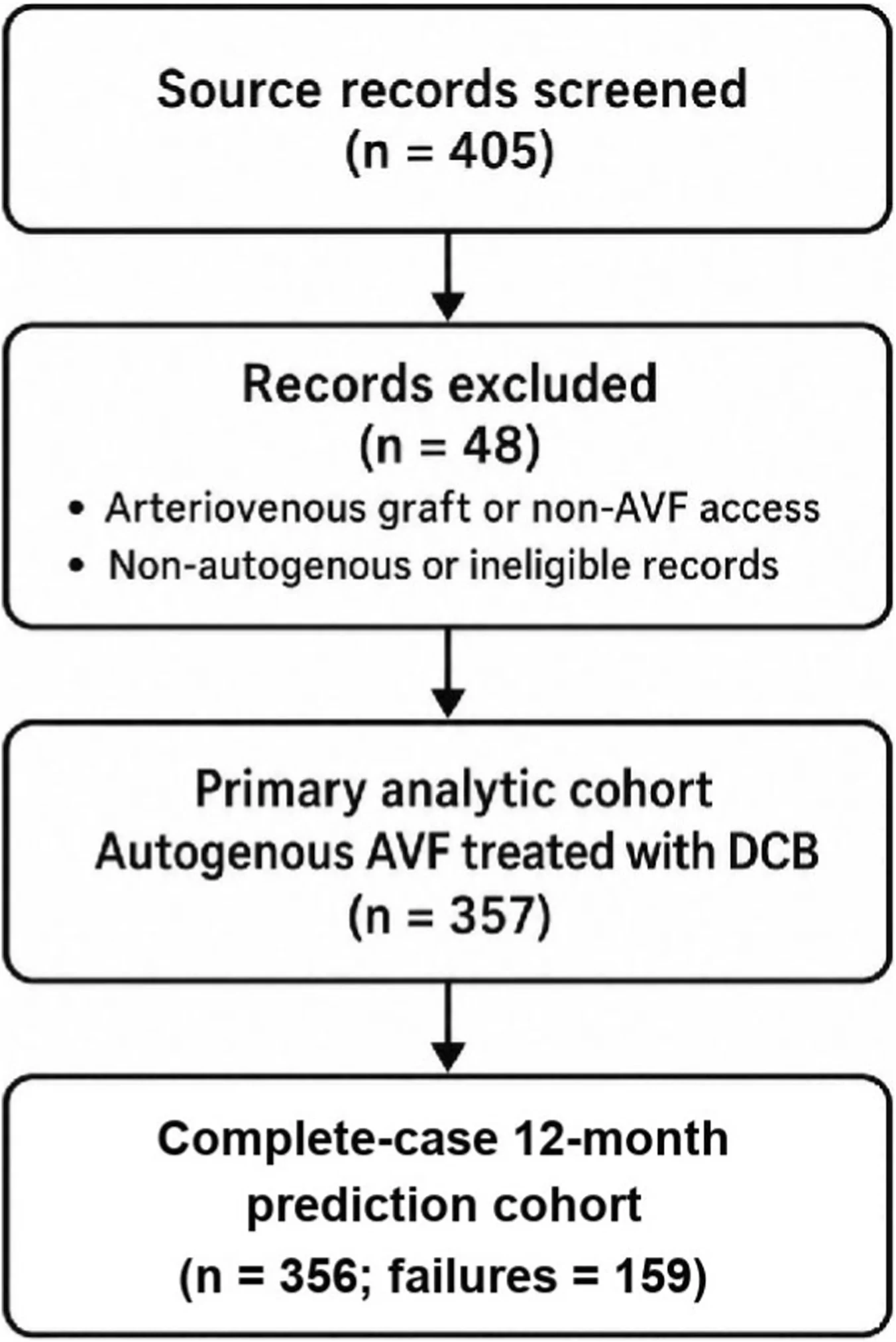
Patient-selection flow diagram. Between February 2023 and May 2026, 405 source records were screened. After 48 arteriovenous-graft or non-autogenous-AVF records were excluded, 357 patients were included in the primary analytic cohort. Of these, 356 patients with complete data were included in the prespecified 12-month prediction analysis, with 159 failures observed within 12 months.

**FIGURE 2:**
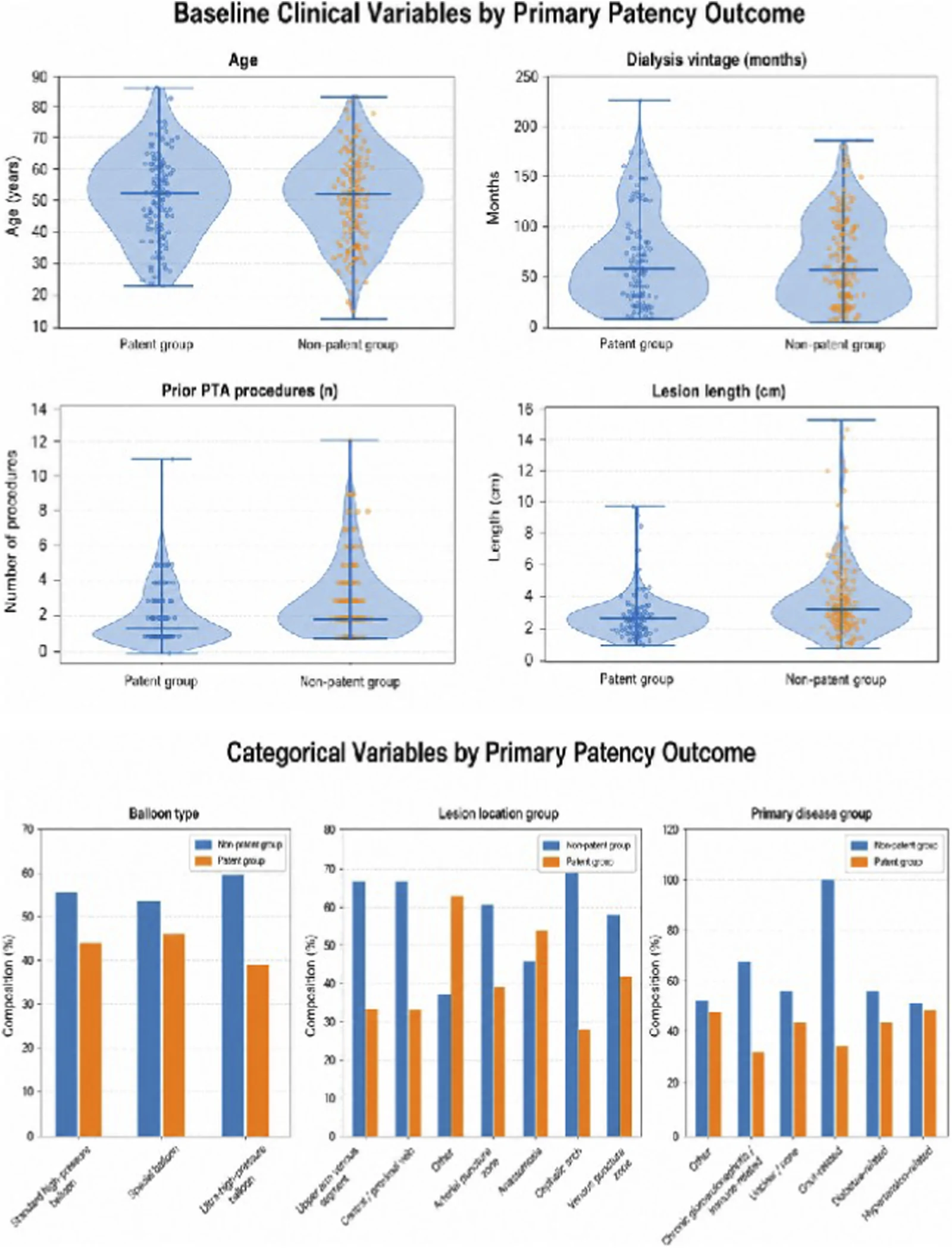
Distribution of baseline clinical and categorical variables by primary-patency outcome. Upper panels show violin plots of age, dialysis vintage, number of prior percutaneous transluminal angioplasty (PTA) procedures and lesion length in the patent and non-patent groups; lower panels show the composition of balloon type, lesion location and primary renal disease by outcome. The non-patent group is characterised by a greater prior intervention burden and longer lesions.

**FIGURE 3:**
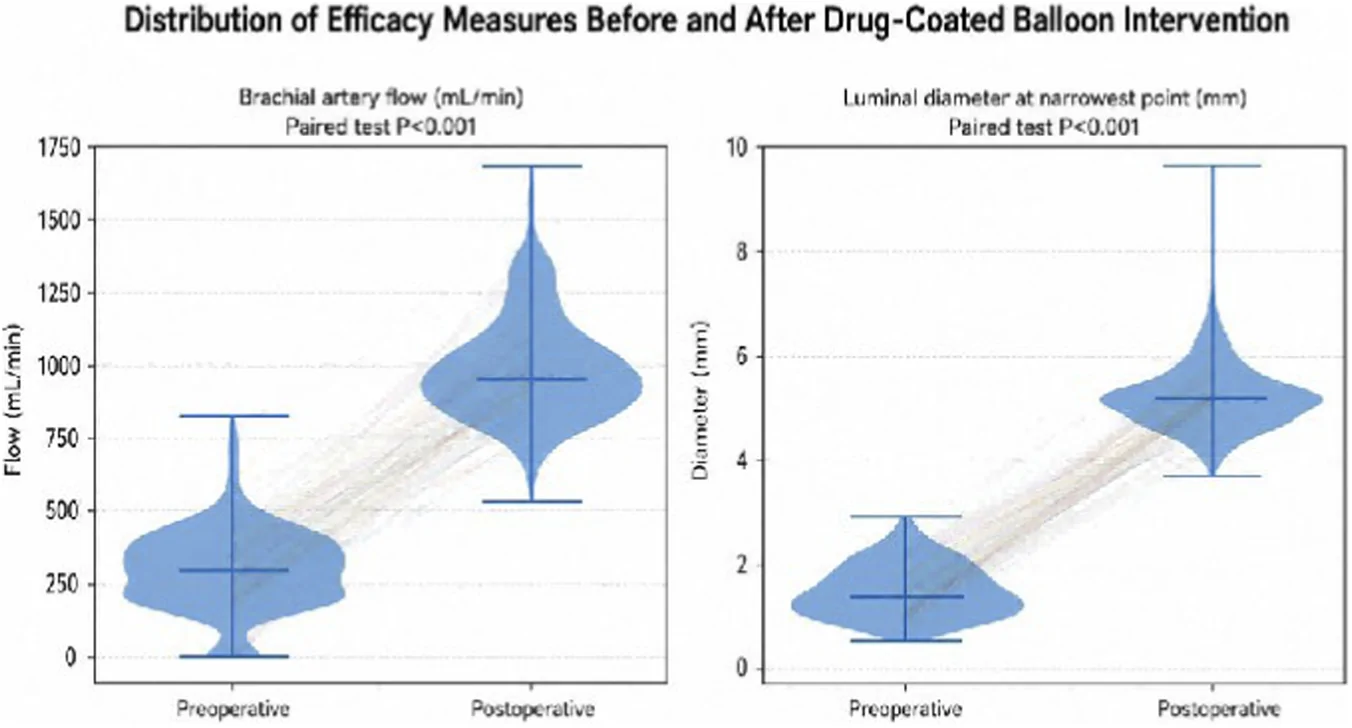
Distribution of efficacy measures before and after drug-coated balloon angioplasty. Violin plots with horizontal medians display the brachial-artery flow volume (left) and the minimal luminal diameter at the narrowest point (right) at the pre-procedural and post-procedural time points; faint connecting lines depict individual patient trajectories. Both measures increased markedly (paired P < 0.001).

**FIGURE 4:**
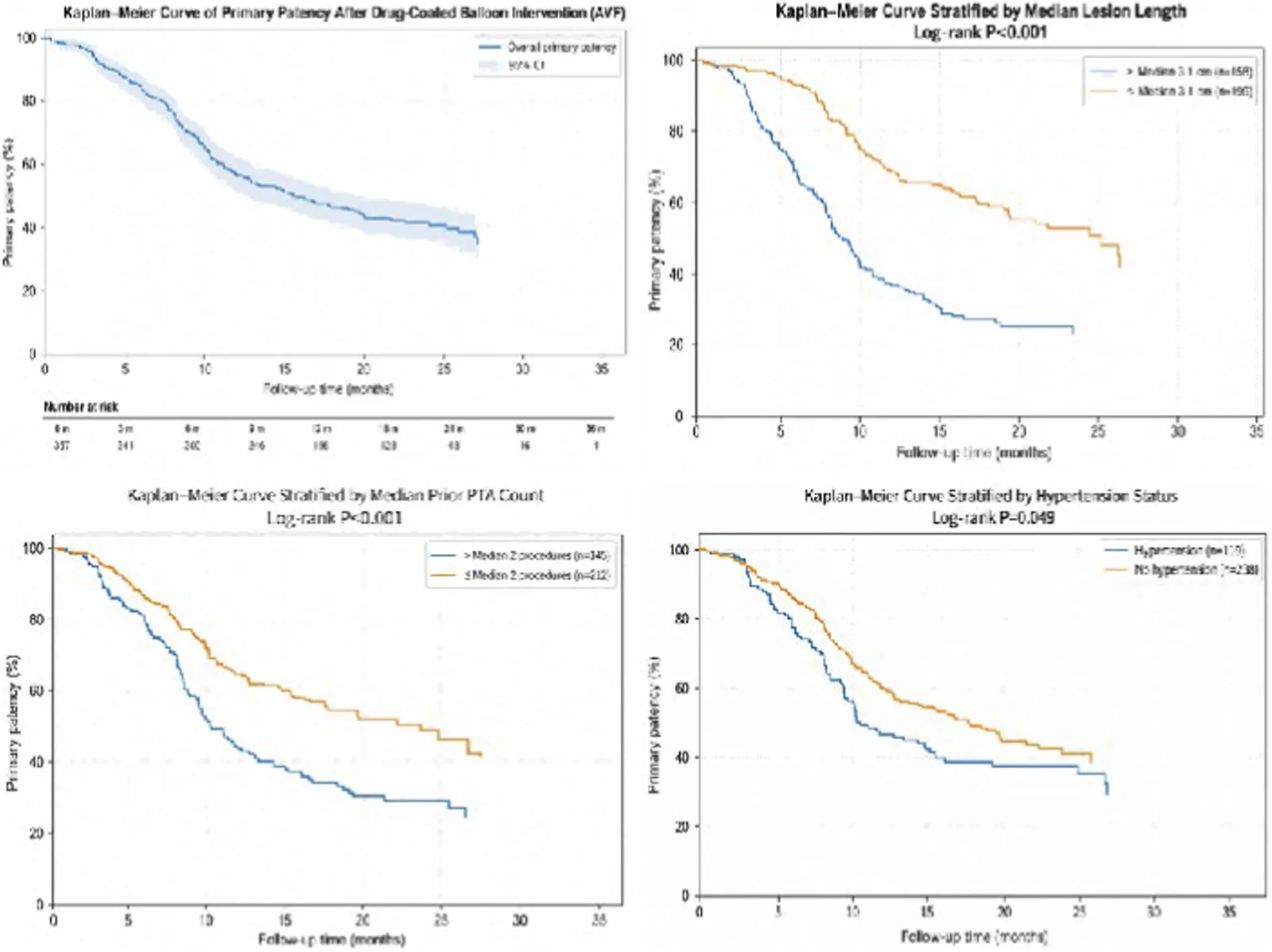
Kaplan–Meier primary-patency curves after drug-coated balloon angioplasty **(A)** Overall primary patency for the whole cohort with the 95% confidence band and number at risk **(B)** Patency stratified by median lesion length (>3.1 cm vs. ≤ 3.1 cm; log-rank P < 0.001) **(C)** Patency stratified by median number of prior PTA procedures (>2 vs. ≤ 2; log-rank P < 0.001) **(D)** Patency stratified by hypertension status (log-rank P = 0.049).

**FIGURE 5:**
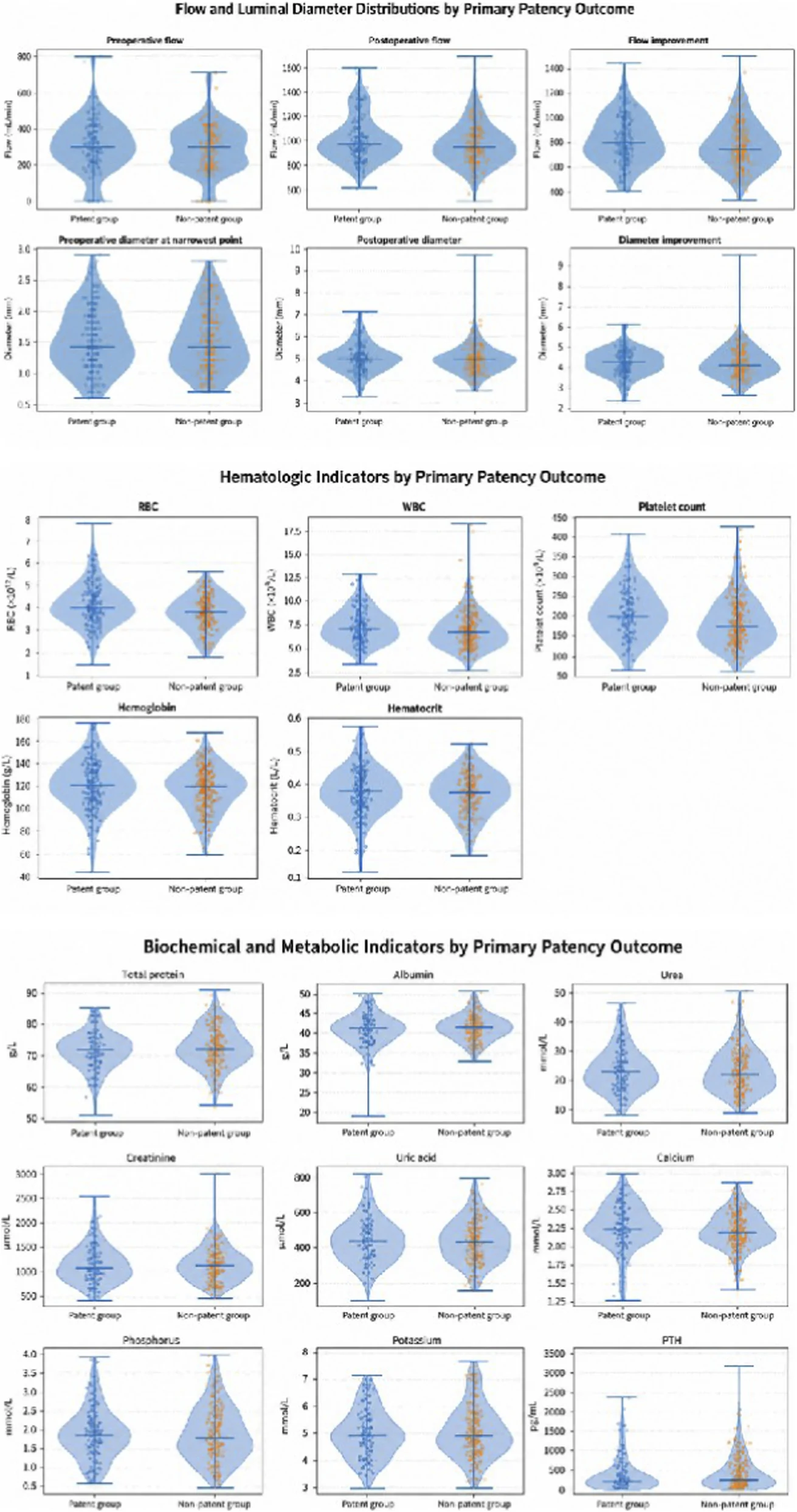
Distributions of haemodynamic and laboratory variables by primary-patency outcome. Violin plots compare the patent and non-patent groups for (top) pre- and post-procedural flow, luminal diameter and their gains (middle) the complete blood count and indices; and (bottom) the serum biochemistry panel. Descriptive differences are most apparent for post-procedural flow, red-blood-cell count and platelet count.

**FIGURE 6:**
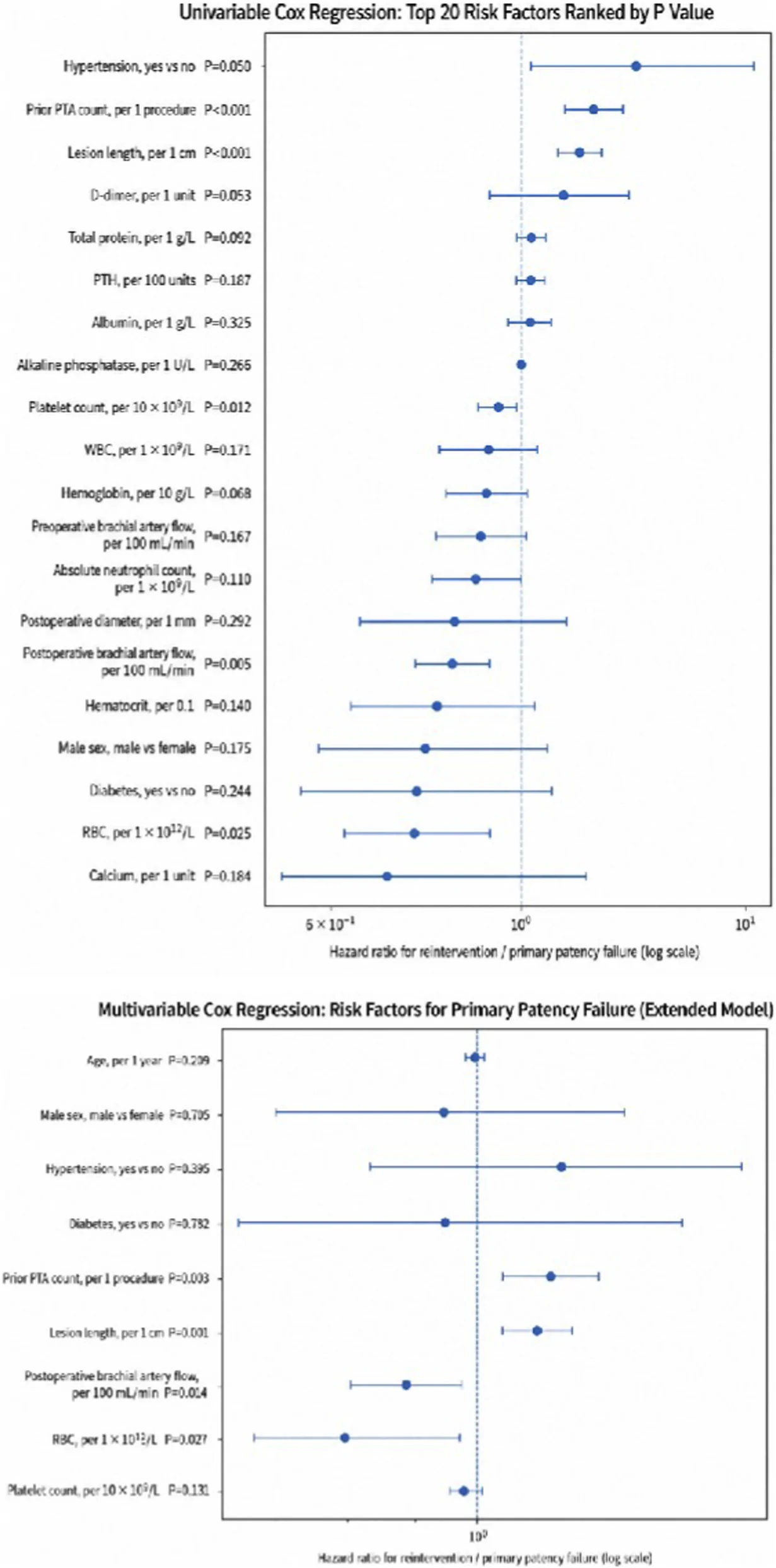
Forest plots of Cox proportional-hazards models for primary-patency loss **(A)** Univariable hazard ratios for the top-ranked candidate variables, ordered by P value **(B)** Adjusted hazard ratios from the extended multivariable model. Squares denote the hazard ratio and horizontal lines the 95% confidence interval; the vertical reference line marks a hazard ratio of 1.0. Prior PTA count and lesion length increase, and post-procedural flow and red-blood-cell count decrease, the hazard of patency loss.

**FIGURE 7:**
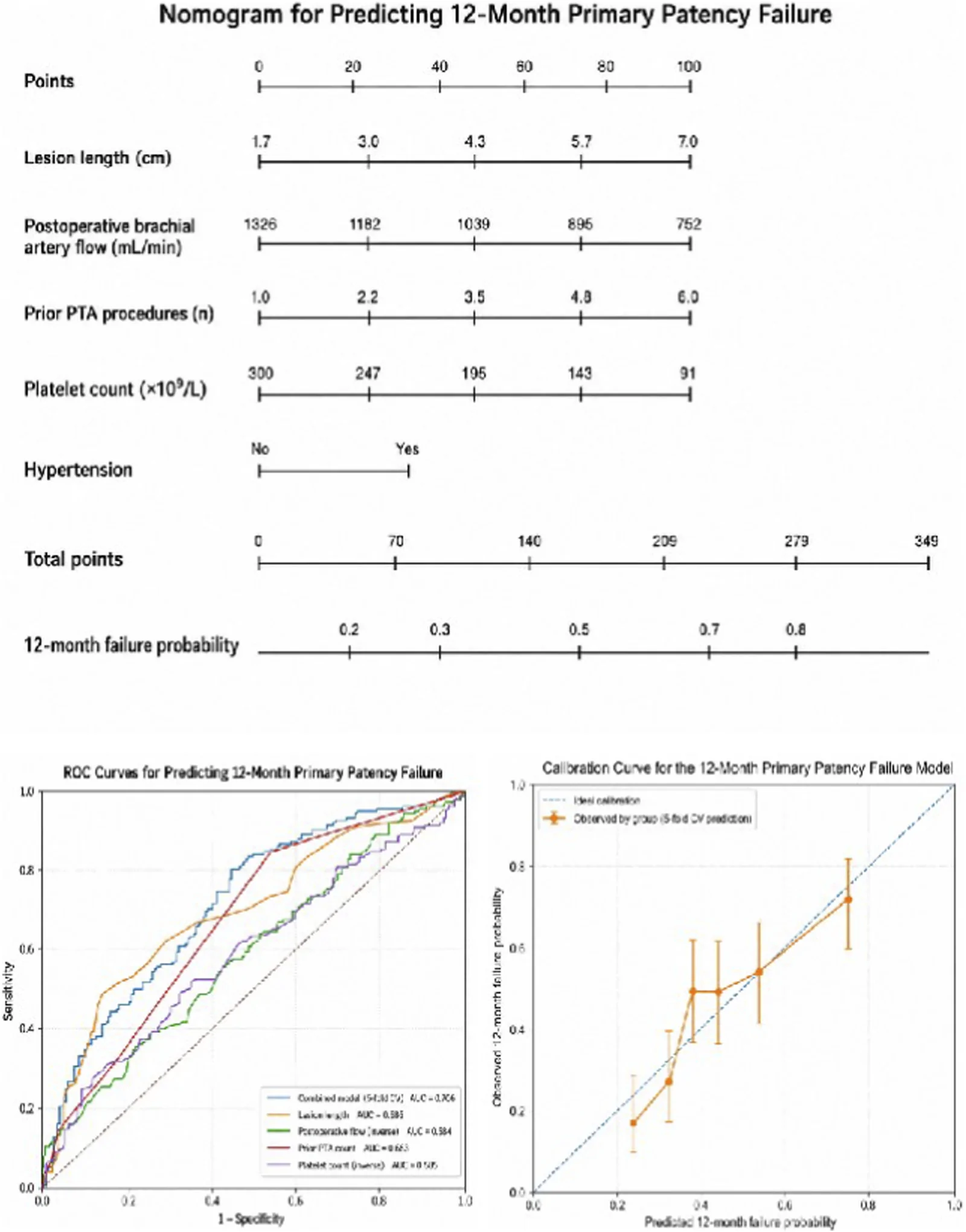
Prediction model for 12-month primary-patency failure. Upper panel: nomogram combining lesion length, post-procedural brachial-artery flow, prior PTA count, platelet count and hypertension; points for each predictor are summed to a total-points axis that maps to the predicted 12-month failure probability. Lower-left panel: receiver-operating-characteristic curves for the combined model (cross-validated AUC 0.706) and individual predictors. Lower-right panel: grouped calibration plot of observed versus predicted failure probability with the line of ideal calibration.

**TABLE 2:** Baseline categorical variables by primary-patency outcome.

Variable	Patent, n (%)	Non-patent, n (%)	χ^2^	P
Sex	​	​	2.17	0.338
Male	82 (53.9)	97 (47.3)	​	​
Female	70 (46.1)	107 (52.2)	​	​
Hypertension	​	​	2.65	0.104
Yes	43 (28.3)	76 (37.1)	​	​
No	109 (71.7)	129 (62.9)	​	​
Diabetes mellitus	​	​	1.11	0.292
Yes	40 (26.3)	43 (21.0)	​	​
No	112 (73.7)	162 (79.0)	​	​
Primary renal disease	​	​	13.02	0.023
Hypertension-related	50 (32.9)	52 (25.4)	​	​
Diabetes-related	27 (17.8)	34 (16.6)	​	​
Glomerulonephritis/immune	19 (12.5)	40 (19.5)	​	​
Gout-related	0 (0.0)	11 (5.4)	​	​
Unspecified/other	56 (36.9)	68 (33.1)	​	​
Lesion location	​	​	8.35	0.214
Anastomosis	42 (27.6)	36 (17.6)	​	​
Arterial cannulation zone	55 (36.2)	87 (42.4)	​	​
Venous cannulation zone	40 (26.3)	56 (27.3)	​	​
Cephalic arch	6 (3.9)	15 (7.3)	​	​
Other	9 (5.9)	11 (5.4)	​	​
Balloon type	​	​	1.08	0.582
Conventional high-pressure	68 (44.7)	86 (42.0)	​	​
Ultra-high-pressure	58 (38.2)	89 (43.4)	​	​
Specialty balloon	26 (17.1)	30 (14.6)	​	​

Data are n (%). Comparisons by Pearson chi-squared test. Bold rows denote P < 0.05.

### 3.2 Immediate haemodynamic and anatomical efficacy

Immediately after the index procedure, brachial-artery flow increased from 288.98 ± 139.37 to 980.92 ± 174.66 mL/min, with a mean increase of 691.94 ± 175.75 mL/min. Minimum luminal diameter increased from 1.52 ± 0.50 to 5.21 ± 0.63 mm, with a mean increase of 3.69 ± 0.65 mm. Both paired comparisons were significant (both *p* < 0.001; Table 3; Figure 3).

**TABLE 3:** Immediate procedural efficacy (primary analytic cohort, n = 357).

Measure	Pre-procedure	Post-procedure	Improvement	P
Brachial-artery flow (mL/min)	288.98 ± 139.37	980.92 ± 174.66	691.94 ± 175.75	<0.001
Minimal luminal diameter (mm)	1.52 ± 0.50	5.21 ± 0.63	3.69 ± 0.65	<0.001

Data are mean ± standard deviation. P values from the paired t-test; the Wilcoxon signed-rank test gave P < 0.001 for both comparisons.

### 3.3 Primary patency over time

Kaplan-Meier primary-patency estimates at 3, 6, 9 and 12 months were 95.24%, 84.03%, 68.90% and 55.40%, respectively (Table 4; Figure 4A).

**TABLE 4:** Kaplan–Meier primary-patency estimates.

Time point	Primary patency (%)	95% CI lower (%)	95% CI upper (%)
3 months	95.24	93.03	97.45
6 months	84.03	80.23	87.83
9 months	68.90	64.10	73.71
12 months	55.40	50.24	60.57

CI, confidence interval. Estimates derived from the Kaplan–Meier survival function for the primary analytic cohort (n = 357; 205 events).

### 3.4 Subgroup patency analyses

Primary-patency curves differed by lesion length (>3.1 cm vs. ≤3.1 cm), prior PTA count (>2 vs. ≤2 procedures) and hypertension status (log-rank *p* < 0.001, *p* < 0.001 and *p* = 0.049, respectively; Figures 4B-D).

### 3.5 Distributions of laboratory and haemodynamic variables by outcome

Red-blood-cell and platelet counts were lower in the non-patent group than in the patent group (*p* = 0.040 and *p* = 0.012, respectively). Post-procedural brachial-artery flow was 966.10 ± 171.86 mL/min in the non-patent group and 1000.90 ± 176.98 mL/min in the patent group (*p* = 0.064; Table 1; Figure 5).

### 3.6 Risk factors for primary-patency loss

Univariable Cox analyses showed associations of prior PTA count, lesion length, post-procedural flow, platelet count and red-blood-cell count with primary-patency loss (Table 5; Figure 6A). In the extended multivariable model (338 patients; 191 events), prior PTA count (adjusted HR 1.129 per procedure, 95% CI 1.042-1.225; *p* = 0.003) and lesion length (adjusted HR 1.105 per cm, 95% CI 1.041-1.172; *p* = 0.001) were associated with a higher hazard of target-lesion reintervention. Higher post-procedural flow (adjusted HR 0.889 per 100 mL/min, 95% CI 0.810-0.977; *p* = 0.014) and red-blood-cell count (adjusted HR 0.804 per 10^12^/L, 95% CI 0.664-0.975; *p* = 0.027) were associated with a lower hazard (Table 6; Figure 6B).

**TABLE 5:** Univariable Cox proportional-hazards analysis for primary-patency loss.

Variable (per unit)	HR	95% CI low	95% CI high	P
Prior PTA count (per procedure)	1.195	1.124	1.269	<0.001
Lesion length (per cm)	1.156	1.102	1.213	<0.001
Post-procedural flow (per 100 mL/min)	0.882	0.808	0.963	0.005
Platelet count (per 10 × 10^9^/L)	0.971	0.948	0.993	0.012
Red-blood-cell count (per 10^12^/L)	0.812	0.677	0.974	0.025
Hypertension (yes vs. no)	1.329	1.000	1.766	0.050
Haemoglobin (per 10 g/L)	0.942	0.883	1.005	0.068
D-dimer (per unit)	1.125	0.998	1.269	0.053
Diabetes (yes vs. no)	0.819	0.585	1.147	0.244
Age (per year)	0.998	0.987	1.008	0.650
Male sex (vs. female)	0.827	0.628	1.088	0.175
Dialysis vintage (per month)	1.000	0.996	1.003	0.781

HR, hazard ratio; CI, confidence interval; PTA, percutaneous transluminal angioplasty. Hazard ratios are expressed per indicated unit increment. Bold rows denote P < 0.05.

**TABLE 6:** Extended multivariable Cox proportional-hazards model for primary-patency loss.

Variable (per unit)	HR	95% CI low	95% CI high	P
Prior PTA count (per procedure)	1.129	1.042	1.225	0.003
Lesion length (per cm)	1.105	1.041	1.172	0.001
Post-procedural flow (per 100 mL/min)	0.889	0.810	0.977	0.014
Red-blood-cell count (per 10^12^/L)	0.804	0.664	0.975	0.027
Platelet count (per 10 × 10^9^/L)	0.981	0.957	1.006	0.131
Hypertension (yes vs. no)	1.151	0.833	1.590	0.395
Diabetes (yes vs. no)	0.946	0.635	1.407	0.782
Age (per year)	0.992	0.980	1.004	0.209
Male sex (vs. female)	0.944	0.699	1.274	0.705

HR, hazard ratio; CI, confidence interval; PTA, percutaneous transluminal angioplasty. Model fitted on 338 patients with 191 events. Bold rows denote P < 0.05.

### 3.7 Prediction model for 12-month primary-patency failure

The clinical logistic model included lesion length, post-procedural brachial-artery flow, prior PTA count, platelet count and hypertension (356 patients; 159 failures within 12 months). The apparent AUC was 0.719 (95% CI 0.667-0.769), and the five-fold cross-validated AUC was 0.706 (95% CI 0.652-0.758). The cross-validated Brier score was 0.220, with a calibration intercept of -0.031 and a calibration slope of 0.852 (Table 7; Figure 7).

**TABLE 7:** Performance of the 12-month primary-patency-failure prediction model.

Metric	Value	95% CI
Patients in 12-month model (n)	356	—
12-month failure events (n)	159	—
Apparent AUC	0.719	0.667–0.769
5-Fold cross-validated AUC	0.706	0.652–0.758
Apparent Brier score	0.216	—
Cross-validated Brier score	0.220	—
Calibration intercept (CV)	−0.031	—
Calibration slope (CV)	0.852	—

AUC, area under the receiver-operating-characteristic curve; CV, cross-validation. The model was internally validated by 5-fold cross-validation. Bold rows denote the principal discrimination metrics.

## 4 Discussion

This retrospective analysis describes patency after DCB angioplasty in a selected recurrent-AVF-stenosis cohort. The inflammatory context of access restenosis is supported by prior vascular-access literature describing injury, neointimal hyperplasia, immune responses and remodeling (MacRae et al., 2016; Brahmbhatt et al., 2016; Lee and Roy-Chaudhury, 2009; Roy-Chaudhury et al., 2006; Roy-Chaudhury et al., 2007; Allon et al., 2013; Kokubo et al., 2009; Remuzzi and Ene-Iordache, 2013). However, neither inflammatory cytokines nor tissue inflammation were measured here. Consequently, lesion length and previous PTA count are anatomical and procedural prognostic variables—not quantified proxies for inflammatory cell death. Associations involving RBC and platelet counts are exploratory hematological observations, not evidence that an inflammatory pathway caused patency failure. Their interpretation requires attention to anemia management, comorbidity, dialysis practice and other confounders (Zhu et al., 2022; Huang et al., 2023; Li et al., 2024; Monroy et al., 2015).

Prior randomized trials and systematic reviews have compared DCBs with conventional angioplasty (Zheng et al., 2021; Lookstein et al., 2020; Holden et al., 2022; Lookstein et al., 2023; Zhao et al., 2024; Trerotola et al., 2020; Pang et al., 2021; Trerotola et al., 2018; Karunanithy et al., 2021). This single-arm cohort has no contemporaneous comparator and cannot attribute the observed time course to a treatment-specific anti-inflammatory effect. Inflammation remains a contextual hypothesis rather than an assessed treatment mechanism. Cohort-derived thresholds and the clinical nomogram should not guide intensified surveillance without external validation (Maleux et al., 2018; Ying et al., 2025).

Limitations include retrospective single-center design; selected recurrent stenosis; heterogeneous lesion preparation and medications; lack of a non-DCB group; potential informative censoring and incomplete follow-up verification; complete-case selection; limited internal model validation; and lack of direct inflammatory biomarkers, cytokine assays, vascular tissue, or molecular measurements. Routine blood counts alone cannot confirm inflammation. The clinical model requires independent validation before application outside this population.

## 5 Conclusion

In this single-center retrospective cohort of patients treated with DCB angioplasty for recurrent autogenous AVF stenosis, previous PTA burden, lesion length, postprocedural flow and red-blood-cell count were associated with target-lesion primary-patency loss. These data support clinical prognostic associations within an inflammation-relevant disease context, but do not demonstrate inflammatory mechanisms, identify validated inflammation biomarkers, or establish a DCB-specific anti-inflammatory effect. External validation and direct measurement of inflammatory indices are needed.

## Data Availability

The de-identified clinical data supporting the findings of this study are available from the corresponding authors upon reasonable request.
